# The relation between gallstone disease and cardiovascular disease

**DOI:** 10.1038/s41598-017-15430-5

**Published:** 2017-11-08

**Authors:** Lai lai Fan, Bai hui Chen, Zhi juan Dai

**Affiliations:** 1 Department of urology, The Second Affiliated Hospital and Yuying Children’s Hospital of Wenzhou Medical University, Zhejiang, P.R. China; 2 Department of endocrinology, The Second Affiliated Hospital and Yuying Children’s Hospital of Wenzhou Medical University, Zhejiang, P.R. China; 3 Department of anesthesiology, The First Affiliated Hospital of Wenzhou Medical University, Zhejiang, P.R. China

## Abstract

Gallstone disease (GD) is a common digestive disorder that shares many risk factors with cardiovascular disease (CVD). CVD is an important public health issue that encompasses a large percentage of overall mortality. Several recent studies have suggested an association between GD and CVD, while others have not. In this report, we present a meta-analysis of cohort studies to assess the association between GD and CVD. We included eight studies published from 1980 to 2017, including nearly one million participants. The pooled relative risk (RR, 95% confidence interval [CI]) from the random-effects model associates with GD is 1.23 (95% CI: 1.17–1.30) for fatal and nonfatal CVD events. The pooled RR from the random-effects model of CVD events in female patients with GD is 1.24 (95% CI: 1.16–1.32). In male GD patients, the pooled RR from the random-effects model for CVD is 1.18 (95% CI: 1.06–1.31). Our meta-analysis demonstrates a substantially increased risk of fatal and nonfatal CVD events among patients with a medical history of GD. We suggest that interested investigators should further pursue the subject. In addition, both male and female patients with GD have a risk of CVD, and women have a higher risk than men.

## Introduction

Gallstone disease (GD) is one of the most common medical problems, exhibiting a prevalence of 10–20% in adults. GD is a common indication for surgical intervention in developed countries^1,2^. GD is also one of the most costly gastrointestinal tract disorders in the world^3^. According to macroscopic appearance and chemical composition, GD is divided into two major types: pigment and cholesterol gallstones^4^.

Cardiovascular disease (CVD) is the leading cause of death globally^5^. CVD is a group of disorders of the heart and blood vessels, including stroke and ischaemic heart disease (IHD)^6^. A number of risk factors of CVD have been identified, such as age^7^, obesity^8^, body mass index (BMI)^9^, low serum high density lipoprotein (HDL) cholesterol levels^10^, diabetes mellitus (DM)^11^, insufficient physical inactivity^12^, smoking^13^, excessive use of alcohol^14^, and elevated blood pressure^15^. These risk factors are also associated with an increased risk of GD.

Recently, many epidemiological studies have reported an association between GD and CVD, while others have found no association. An analysis published in 2016 by Zheng *et al*.^16^ that included five articles suggested that GD was associated with an increased risk of coronary heart disease (CHD). However, the analysis was hampered by a degree of high heterogeneity. Neither heterogeneity analysis nor further subgroup analysis was performed. Finally, these researchers’ analysis was limited to CHD, rather than to CVD. Therefore, we performed a meta-analysis of cohort studies to further explore a possible association between GD and CVD.

## Methods

### Search strategy

In October 2016, we searched PubMed and EMBASE for studies describing the association between GD and CVD. We updated the search in June 2017 to verify that our study was based on the most current data. We also checked the references of included studies and reviews. Only papers issued in the English language were considered. The search focused on six medical subject headings terms and key words: gallstone disease, stroke, coronary heart disease, myocardial infarction (MI), ischaemic heart disease, and cardiovascular disease. The logical operator “and” was used to combine search terms.

### Study selection

Literature eligibility was assessed by two investigators independently. Discordant conclusions were settled by consensus. Inclusion criteria were as follows: (1) the study was a cohort study; (2) the authors reported data from an original, peer-reviewed study (i.e., not review articles or meeting abstracts); and (3) the authors reported risk estimates of the association between GD and CVD. When an article included multiple publications, we included the article with the longest follow-up years or the largest number of incident cases. We qualified articles for further examination by performing an initial screen of identified titles and abstracts, followed by full-text review.

### Data extraction

The following information was extracted from the included studies: study name, authors, publication year, region, study population, study design, age range, percentage female patients, years of follow-up, sample size, outcomes, data collection, assessment of GD, adjusted relative risk (RR, 95% confidence interval [CI]) and confounder adjustment. The primary clinical outcome of the study was a combined endpoint including fatal and nonfatal CVD events. If the information was unavailable from the report, we attempted to collect relevant data by corresponding with the authors. We utilized the Newcastle - Ottawa Quality Assessment Scale (NOS)^17^ to evaluate the quality of included studies with consideration of the following aspects: selection, comparability and exposure.

### Data synthesis and analysis

The fully adjusted RR was used to estimate the association between GD and CVD. Forest plots were created to visually assess the RRs and corresponding 95% CIs across studies. In the forest plots, each study as well as its summary effect was depicted as a point estimate bounded by a confidence interval. This representation showed whether the effects for all studies were consistent or whether they varied substantially from one study to the next. RR > 1 and 95% CI excluding 0 meant a positive correlation^18^. Heterogeneity across studies was assessed by the Cochrane Q statistic (significance level of p < 0.10) and the I^2^ statistic (ranges from 0–100%, with lower values representing less heterogeneity)^19^. The RRs were pooled using random-effects models^20^.

Pre-specified subgroup analyses were performed to examine the impacts of various study characteristics, including region, years of follow-up, sample size, rate of CVD events and the degree of adjustment for the most important confounders. A sensitivity analysis was conducted to assess the influence of each individual study on the summary risk estimate using the trim and fill method^21^. Remaining studies were reanalysed following the omission of one study at a time. Finally, the potential publication bias was examined by visual inspection of the funnel plot and the result of Egger’s test (p < 0.10)^22^. A roughly symmetrical funnel plot suggested no publication bias^23^. All analyses were performed using STATA version 14.1 (Stata Corp, College Station, Texas). A p-value < 0.05 was considered statistically significant, except where otherwise specified^24^.

## Results

### Literature search

A total of 566 articles were retrieved in the initial search. Of these, 20 duplicate articles were excluded. After a first round of screening based on titles and abstracts, 12 articles remained for further review. After comprehensive full-text examination, four articles were excluded as they were reviews. Ultimately, eight articles^16,25–31^ were eligible for analysis (Table 1).

**Table 1:**
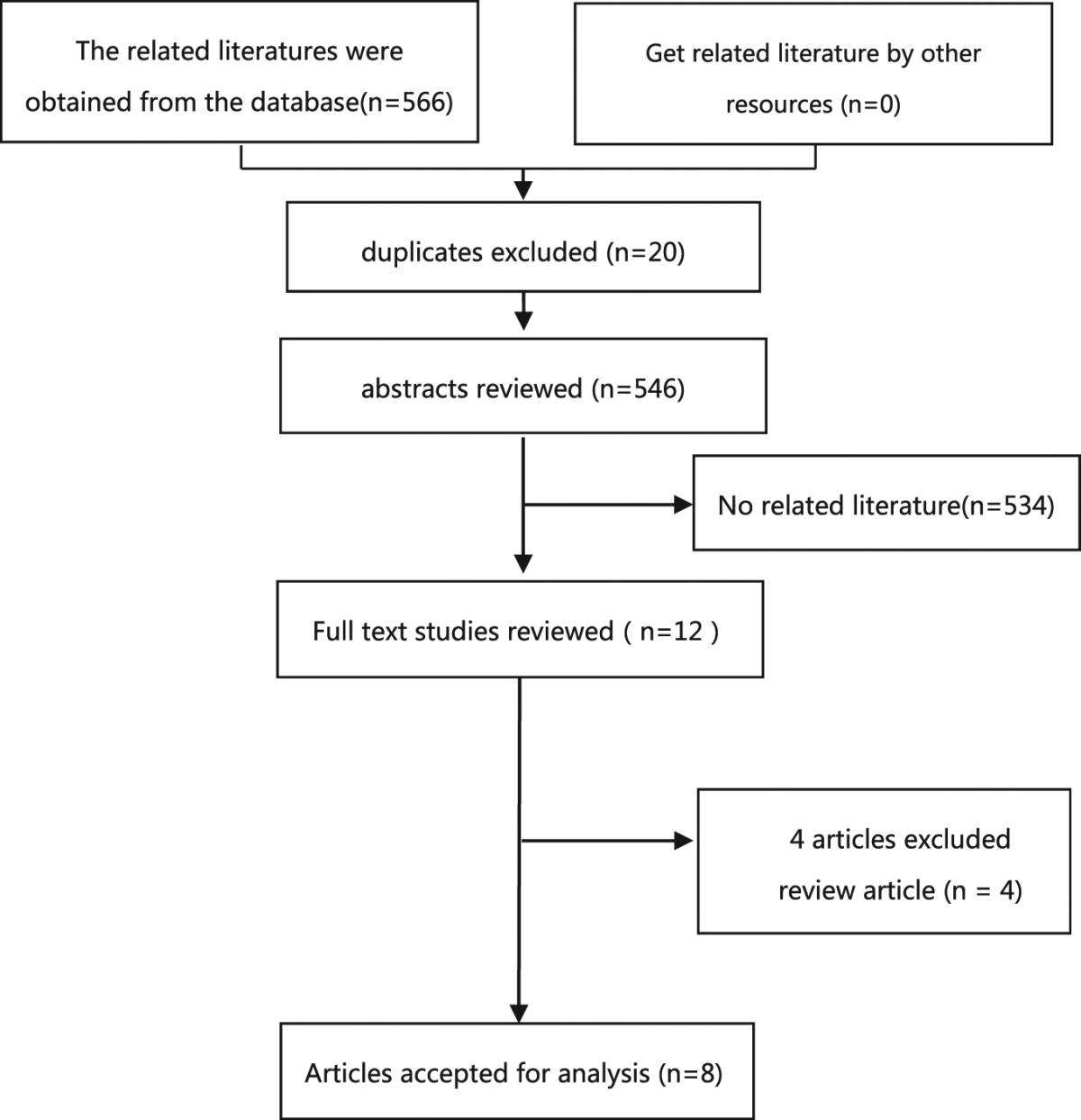
Flow chart of the meta-analysis of the relation between gallstones and cardiovascular disease.

### Study characteristics

There were 11 retrospective cohort studies among the eight articles: one article^16^ included three cohort studies, and another article^25^ included two cohort studies. The characteristics of the 11 studies among the eight articles are displayed in Table 2. Five studies were questionnaire-based, and six studies were reviews of hospital records. Five studies specifically reported results on CHD; two studies reported CVD mortality; one study reported IHD; one study reported stroke; and two studies reported multiple outcomes. The assessment of GD varied across studies: one study used definite hospital diagnosis; two studies relied on ICD codes; two studies relied on evidence of a cholecystectomy or a definite hospital diagnosis; one study relied on evidence of a cholecystectomy and imaging diagnosis; three studies relied on evidence of a cholecystectomy, a definite hospital diagnosis, or an imaging diagnosis; and one study relied on evidence of a cholecystectomy, a definite hospital diagnosis, an imaging diagnosis, or postmortem pathologic examination. Eight studies were published after 2010. With regard to the study region, three studies were published in Asia, seven in the US, and one in Germany. Three studies included only female patients. Two studies included only males. Follow-up duration ranged from 6 to 30 years. The age range of participants in most studies included young and middle-aged patients, and the upper limit for one study was 80^28^. The maximum sample size was 487,373^27^, and the minimum sample size was 605^26^. The average sample size was 107,057. Adjustment for potential confounding factors varied among studies. Most risk estimates were adjusted for age and gender. The study types of included studies were all retrospective cohort studies.

**Table 2:** Characteristics of studies included in the meta-analysis of the relation between gallstones and cardiovascular disease.

Author, year	Zheng *et al*.,			Wirth *et al*., 2015	Lv *et al*., 2015	Wei *et al*., 2014	Olaiya *et al*., 2013	Ruhl *et al*., 2011	Grimaldi *et al*., 1993	Bortnichak *et al*.,	
	2016(1)	2016(2)	2016(3)							1985(1)	1985(2)
region	US	US	US	German	China	China	China	US	US	US	
Study population	NHS	NHSII	HPFS	EPIC	CKBS	NHIRD	NHIRD	NHANES	CRIC	FHS	
Study design	Retrospective cohort	Retrospective cohort	Retrospective cohort	Retrospective cohort	Retrospective cohort	Retrospective cohort	Retrospective cohort	Retrospective cohort	Retrospective cohort	Retrospective cohort	
Age(Y)	30–55	25–42	40–75	35–65	30–79	NA	18–80	53.2	NA	28–62	
Female (%)	100	100	0	59	59.1	50.7	56.2	52	NA	0	100
Follow–up (Y)	Up to 30	Up to 22	Up to 22	8.2	7.2	NA	6	14.3	20	26	
Sample size	20,316	1,18,146	45,152	46,486	4,87,373	4,06,536	34,275	14,228	605	2,127	2,381
Outcome	CHD	CHD	CHD	MI and stroke	IHD	Stroke	CVD, Stroke, CHD, HF	CVD mortality	CVD mortality	CHD	
Data collection	questionnaires			questionnaires	questionnaires	inpatient fifiles	inpatient claims	records	medical records	medical records	
Assessment of GD	unremoved gallstones, cholecystectomy	cholecystectomy, a diagnosis of gallstones from a physician, radiography or ultrasonography		diagnosed with gallstones, cholecystectomy	diagnosed with GD by a doctor	ICD-9-CM	ICD-9-CM.	ultrasound-documented gallstones, evidence of a cholecystectomy	oral cholecystograms, diagnosed with GD, cholecystectomy	history of gallbladder surgery, abnormal gallbladder x-ray, a definite diagnosis of gallbladder disease, postmortem pathologic examination	
Confounder Adjustment	Age, BMI, MI, family history, smoking, alcohol, daily cholesterol intake, daily energy intake, physical activity, race, marital status, post–menopausal hormone replacement, Alternative Health, Eating Index Score, hypercholesterolemia, HTN, DM, regular aspirin use			age, sex, study center, educational achievement, physical activity, smoking, alcoholism, BMI, WC, HTN, HL	age, sex, education, level of education; marital status; alcohol consumption; smoking; physical activity; intake of red meat, fresh fruits, vegetables; HTN and DM; family history of heart attack, BMI, WC, menopausal status, digestive system diseases	age, sex, history of HTN, DM, CHD, HF, HL	Age, sex, peripheral vascular disease, COPD, DM, HDL, HTN, alcoholism, chronic liver disease, and anemia	age, sex, race, education, BMI, WHR, glucose status, total serum cholesterol, HDL, smoking, drinking, caffeine, physical activity, CRP, SBP, DBP	age, sex, age-sex interaction, BMI, cholesterol, DM.	Age, sex, DM, left ventricular hypertrophy, TC, length of follow-up, SBP, Framingham Relative Weight, smoking, cholecystectomy	
Relative risk	1.15	1.33	1.11	1.24)	1.23	1.29	1.32	1.30	1.10	1.60	0.72
(95% Cl)	(1.10–1.21)	(1.17–1.51)	(1.04–1.20)	(1.02–1.50	(1.17–1.28)	(1.26–1.32)	(1.22–1.43)	(0.87–2.0)	(0.6–2.3)	(1.13–2.28)	(0.42–1.22)
Quality assessment (NOS)	Selection:4	Selection:4	Selection:4	Selection:4	Selection: 3	Selection: 4	Selection: 4	Selection: 4	Selection: 4	Selection: 4	
	Comparability: 2	Comparability: 2	Comparability: 2	Comparability: 2	Comparability: 2	Comparability: 2	Comparability: 2	Comparability: 2	Comparability: 2	Comparability: 2	
	Outcome: 2	Outcome: 2	Outcome: 2	Outcome: 2	Outcome: 2	Outcome: 2	Outcome: 2	Outcome: 3	Outcome: 3	Outcome: 2	

NHS: The Nurses’ Health Study; NHSII: The Nurses’ Health Study II; HPFS: The Health Professionals Follow-up Study; HTN: hypertension; EPIC: The European Prospective Investigation into Cancer and Nutrition; CKBS: The China Kadoorie Biobank Study; NHIRD: The National Health Insurance Research Database; NHANES: National Health and Nutrition Examination Survey; CRIC: Gila River Indian Community; FHS: Framingham Heart Study; ICD-9-CM: International Classification of Diseases, Ninth Revision, Clinical Modification; WC: waist circumference; HL: hyperlipidemia; HF: heart failure; COPD: chronic obstructive pulmonary disease; WHR: waist-to-hip ratio; CRP: C-reactive protein; SBP: systolic blood pressure; DBP: diastolic blood pressure; TC: total cholesterol.

### GD and risk of CVD

**Figure 1:**
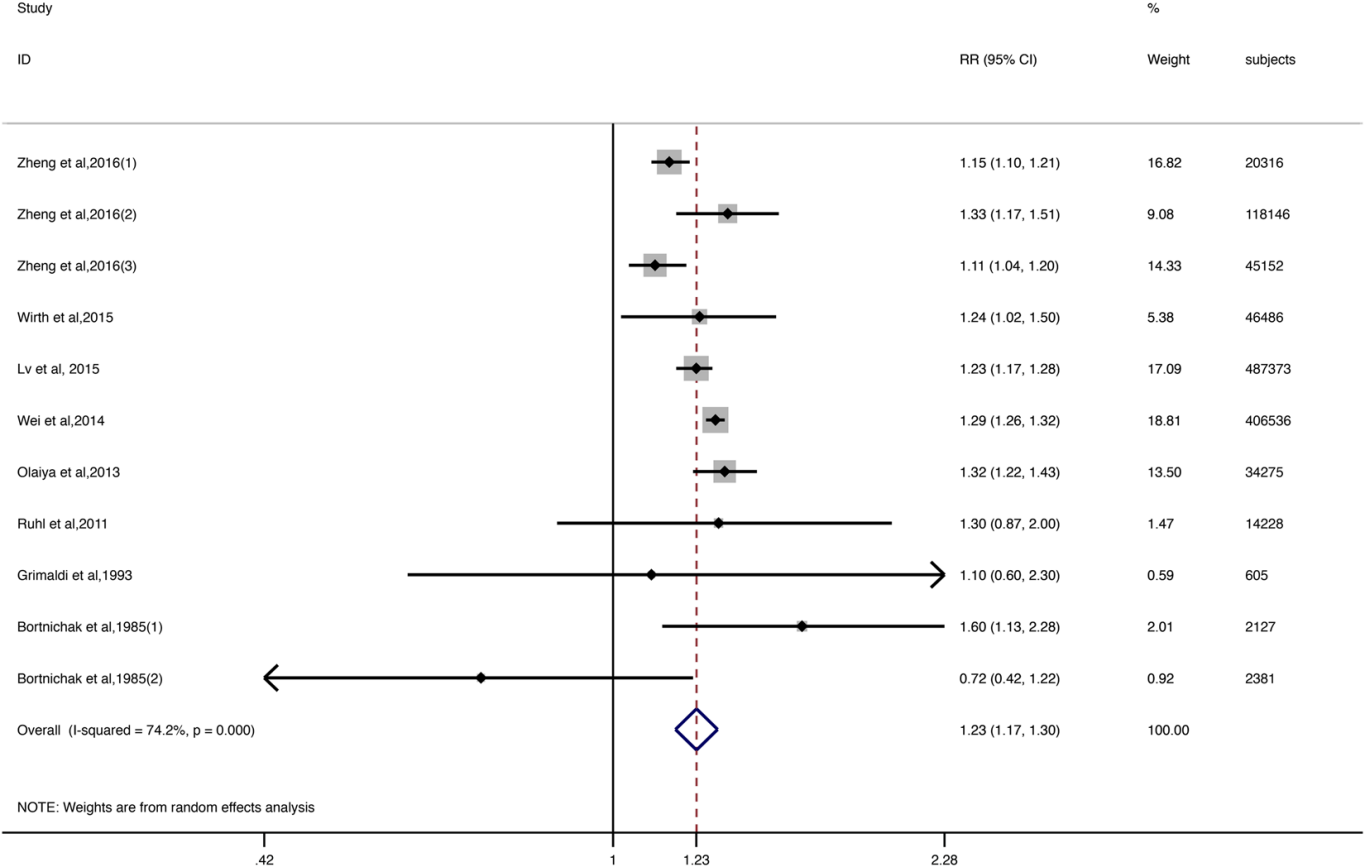
The squares and horizontal lines correspond to the study-specific RR and 95% CIs. The area of the squares reflects the study-specific weight. Weights are from random effects analysis. The diamond represents the pooled RR and 95% CI.

The majority of studies reported a positive association, but the RRs reported by three articles were not statistically significant^25,26,29^. Patients with GD had a 23% higher risk of CVD than the patients in the control groups [95% CI = 1.17–1.30, Fig. 1]. We detected substantial heterogeneity among studies (I^2^ = 74.2%; p < 0.000).

### Subgroup and sensitivity analyses

We conducted subgroup analyses by length of follow-up, sample size, region, rate of CVD events, and the degree of adjustment for the most important confounders (Table 3). The subgroups of ≦10 years follow-up (I^2^ = 13.4%, RR: 1.26, 95% CI: 1.20–1.31), ≦50,000 participants (I^2^ = 20.8%, RR: 1.27, 95% CI: 1.24–1.31), CVD mortality (I^2^ = 0%, RR: 1.05, 95% CI: 0.94–1.16), and incomplete adjustment (I^2^ = 39%, RR: 1.30, 95% CI: 1.21–1.39) showed a marked decrease in heterogeneity. We observed a non-significant association between GD and fatal CVD events, but this result was not reliable due to a lack of data (only three studies reported the fatal CVD events). We therefore speculated that heterogeneity might result from years of follow-up, number of participants and the degree of adjustment for the most important confounders.

**Table 3:** Stratified analyses of the risk of cardiovascular disease among gallstones patients.

Group	RR(95%CI)	Reports	I^2^ (%)	P_(heterogeneity)_
**Region**				
**US**	1.18(1.09,1.29)	7	52.3	0.050
**Asia**	1.27(1.23,1.32)	3	50.3	0.133
**Follow-up(year)**				
**>10**	1.18(1.09,1.29)	7	52.3	0.050
**≦10**	1.26(1.20,1.31)	3	13.4	0.315
**sample size**				
**>50,000**	1.20(1.10,1.30)	7	66.7	0.006
**≦50,000**	1.27(1.24,1.31)	4	20.8	0.285
**CVD events**				
**Morbidity**	1.23(1.17,1.30)	9	79.3	0.000
**Mortality**	1.05(0.94,1.16)	3	0	0.563
**Adjustment**				
**Complete**	1.19(1.13,1.26)	6	54.2	0.053
**Incomplete**	1.30(1.21,1.39)	5	39.0	0.161

**Figure 2:**
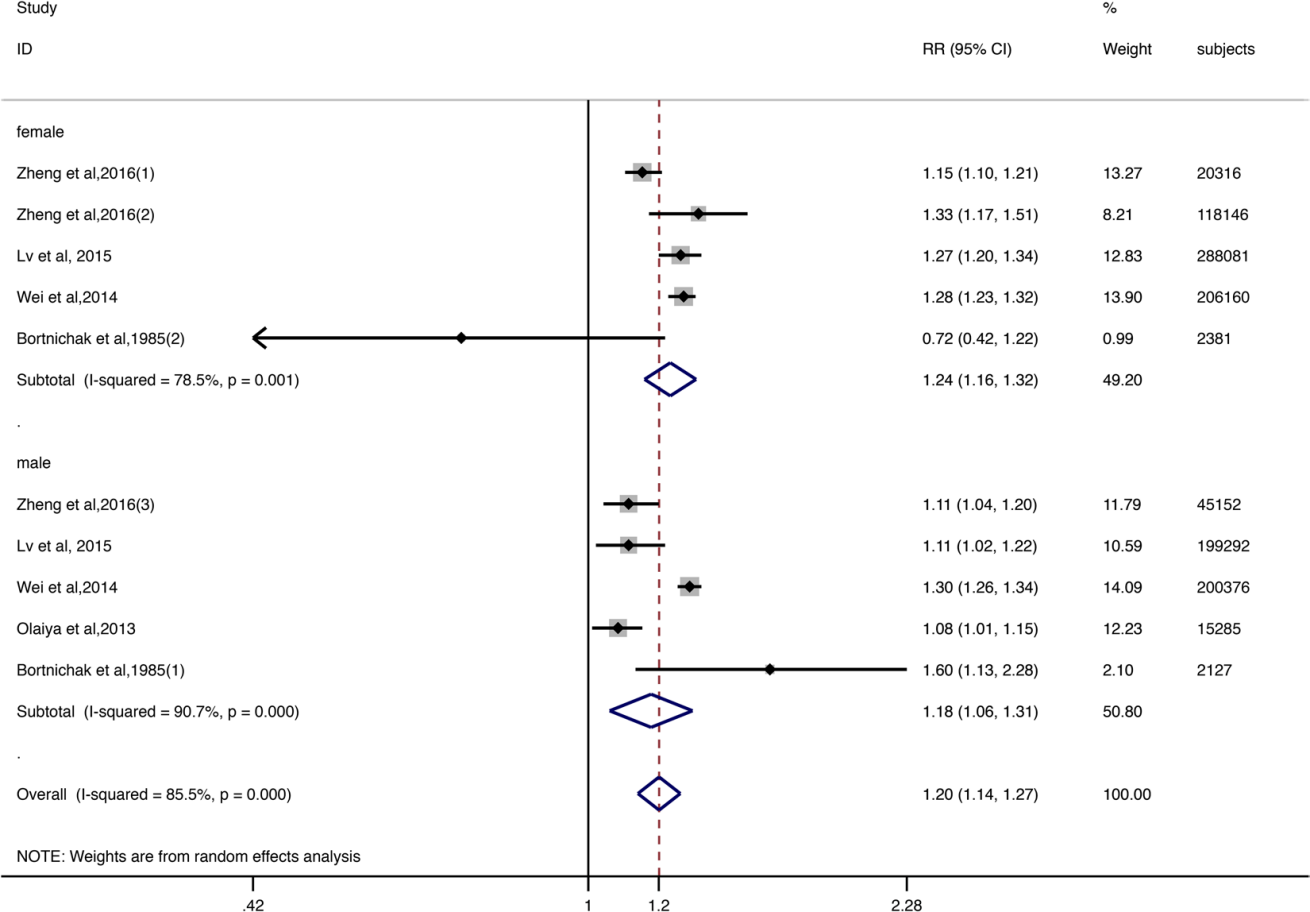
The squares and horizontal lines correspond to the study-specific RR and 95% CIs. The area of the squares reflects the study-specific weight. Weights are from random effects analysis. The diamond represents the pooled RR and 95% CI.

There were five articles with eight studies reporting the relative risk for males and/or females. One study reported a RR < 1.00, but this estimate was not statistically significant. Pooled RR from the random-effects model for women was 1.24 (95% CI: 1.16–1.32, I^2^ = 78.5%, Fig. 2). The pooled RR from the random-effects model for men was 1.18 (95% CI: 1.06–1.31, I^2^ = 90.7%, Fig. 2). Both sexes with GD had a risk of CVD, but the risk for women was higher than that of men.

**Figure 3:**
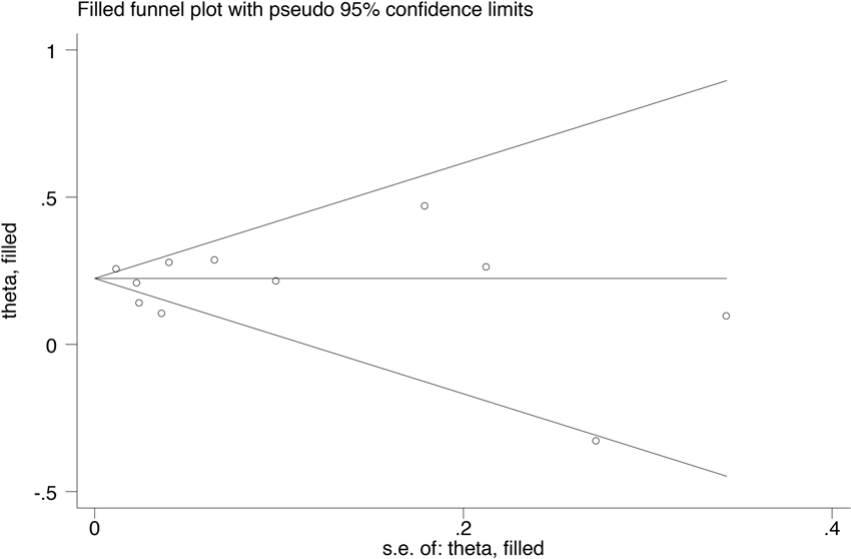
Circles represent identified studies.

A sensitivity analysis of omitting one study at a time showed no substantial change in the results. The trim and fill method showed no trimming, and the data were unchanged (Fig. 3).

### Cholecystectomy and risk of CVD

Wirth *et al*.^31^ and Ruhl *et al*.^29^ reported that cholecystectomy increased the risk of CVD, with a rate of surgery of 66.2% and 74.6% among GD patients. The RRs were 1.32 (95% CI: 1.05–1.65) and 1.3 (95% CI: 1.1–1.6), respectively. Olaiya *et al*.^28^ and Zheng *et al*.^23^ suggested a trend towards no differences among groups, but there were insufficient data to perform a statistical analysis.

### Publication bias

**Figure 4:**
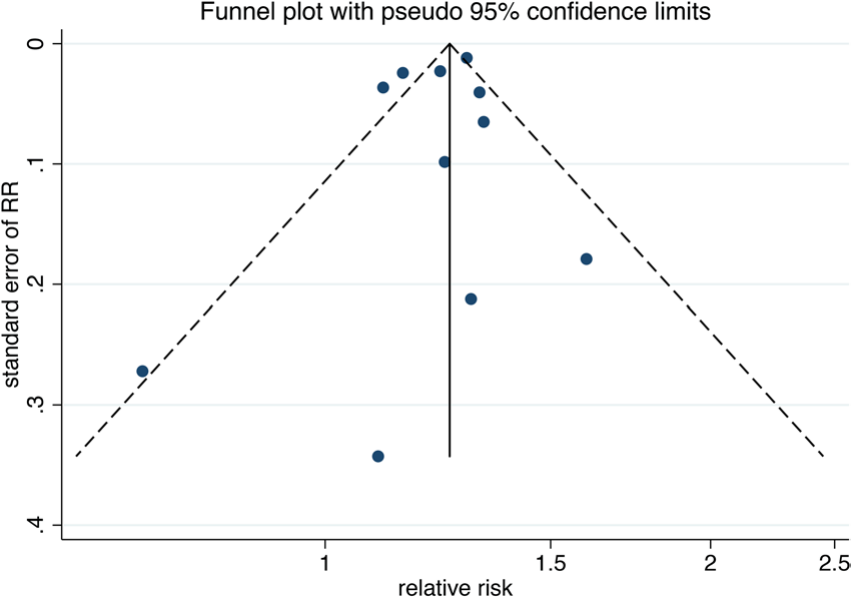
Circles represent identified studies.

There was no publication bias according to the visual inspection of the funnel plot (Fig. 4) and the result of Egger’s test (p = 0.467).

## Discussion

In this meta-analysis comprising approximately one million participants, we demonstrate that a history of GD gives a 1.23-fold increased risk of CVD. We also demonstrate that women may have a higher risk of CVD than men. In addition, patients undergoing cholecystectomy may have a higher risk of CVD than GD patients without surgical treatment, but the data are insufficient to draw a statistically significant conclusion.

Most of the studies attribute both GD and CVD to common risk factors. However, the RRs collected from included studies were all adjusted for these common risk factors, such as age, obesity, BMI, diabetes, hypertension, unhealthy diet and physical inactivity. All but two articles^16,27^ show a decline in RR after adjustment, but the results still were significant, these two articles suggest that hypertension, obesity and diabetes mellitus are protective factors. Two studies^28,30^ suggest that younger patients are at higher risk than older patients, but that the elderly in general tend to have more risk factors. Taken together, these results suggest aetiologies apart from the known common risk factors. Cholesterol accumulation is a major feature of both GD and atherosclerosis. The association between GD and CVD may due to a shared metabolic pathway involving cholesterol and other pathophysiological features. Low HDL level is known to increase risk of CVD morbidity and mortality^32^ and has been shown to play a role in the development of GD^33^. One study suggests that insulin-like growth factor one (IGF-1) is involved in gallbladder emptying and may have an anti-atherosclerotic effect, which suggests that low plasma levels of IGF-1 may result in both GD and CHD^34^. Oxidative stress also plays an important role in the development of GD^35^ and has been implicated in the pathogenesis of CVD as well^36^.

Many studies indicate that the gut microbiota influences host health. A recent study suggests that altered composition of gut microbiota increase the risk of CVD by derived signalling molecules^37^, and GD is related to microbiota dysbiosis in the gut and biliary tract^38^. Mounting evidence suggests that non-alcoholic fatty liver disease (NAFLD) is a risk factor for IHD^39^. Additionally, a recent study shows an association between GD and NAFLD^40^, and preliminary evidence suggests that GD is associated with more severe liver damage in NAFLD patients^41,42^. Although the mechanisms have not been fully elucidated, these studies suggest new avenues for prevention and treatment.

Traditionally, CVD has been thought of as a male disease. According to our study, however, women with GD may have a higher risk of CVD than men. The explanation for this phenomenon is unknown, but we speculate that it may be related to the following factors. Low HDL levels contribute to the development of GD^33^ and CVD, peak total cholesterol levels occur later in men than in women, and HDL levels decrease in postmenopausal women. Diabetes increases the risk of GD^43^ and death from CHD^44^, and the incidence of diabetes in women is higher than in men. Elderly women with CHD are more likely to suffer from metabolic syndrome^44^. Low socioeconomic status increases the risk of CVD^45^ and GD^46^.

There are two distinct points of view regarding whether cholecystectomy increases the risk of CVD in GD patients. Wirth *et al*.^31^ and Ruhl *et al*.^29^ suggest that cholecystectomy increases the risk, while Olaiya *et al*.^28^ and Zheng *et al*. [23] find no significant difference. We agree with the former viewpoint, though there are not enough data to support this conclusion. Our reasons are as follows: cholecystectomized mice have elevated serum levels of very low-density lipoprotein^47^; cholecystectomy may impact lipid and glucose metabolism^48,49^; gallbladder-related hormones have a beneficial effect on metabolic syndrome^50^; and cholecystectomy changes bile flow to the intestine and therefore alters the microbiota between bile acids and the intestine^51^. More studies are needed to establish a connection more firmly.

Several limitations of this meta-analysis should be acknowledged. First, we find substantial heterogeneity across studies, possibly arising from years of follow-up, number of participants and the degree of adjustment for the most important confounders. Second, the meta-analysis is restricted to English-language publications, and the possibility of unpublished reports is not yet identified. Third, although the assessment of GD varies across these cohort studies, most studies include evidence of a cholecystectomy or a definite hospital diagnosis. Therefore, we do not believe that differences in assessments will reverse the results. Fourth, the varying degree of confounder adjustments across the individual studies hampers a systematic assessment of the impact of known risk factors on the outcome of interest. Finally, the observational retrospective design does not allow for establishing causality. The strengths of our study include the following: we performed a comprehensive systematic search for eligible studies; literature eligibility was assessed by two investigators independently; we included sufficient numbers of participants with ample follow-up time; no significant publication bias was found; and the sensitivity analysis showed no substantial change in the results.

## Conclusions

Our meta-analysis demonstrates a substantially increased risk of CVD among patients with a medical history of GD. We suggest that interested investigators should further pursue the subject. We show that the women may have a higher risk of CVD than men and that cholecystectomy may increase the risk of CVD. Further research is warranted.
